# Antenatal corticosteroids and preterm offspring outcomes in hypertensive disorders of pregnancy: A Japanese cohort study

**DOI:** 10.1038/s41598-020-66242-z

**Published:** 2020-06-09

**Authors:** Takafumi Ushida, Tomomi Kotani, Masahiro Hayakawa, Akihiro Hirakawa, Ryo Sadachi, Noriyuki Nakamura, Yoshinori Moriyama, Kenji Imai, Tomoko Nakano-Kobayashi, Fumitaka Kikkawa

**Affiliations:** 1 Department of Obstetrics and Gynecology, Nagoya University Graduate School of Medicine, Nagoya, Japan; 2 Division of Neonatology, Center for Maternal-Neonatal Care, Nagoya University Hospital, Nagoya, Japan; 3 Department of Biostatistics and Bioinformatics, Graduate School of Medicine, The University of Tokyo, Tokyo, Japan; 4 Neonatal Research Network of Japan, Tokyo, Japan

**Keywords:** Preterm birth, Neonatology, Paediatric research

## Abstract

To estimate whether antenatal corticosteroids (ACS) improve short- and long-term preterm offspring outcomes in singleton pregnancies complicated by hypertensive disorders of pregnancy (HDP) similar to pregnancies without HDP. This population-based retrospective study was conducted based on an analysis of data collected by the Neonatal Research Network of Japan on 21,014 singleton neonates weighing ≤1,500 g between 24 and 31 weeks’ gestation during 2003–2016. Logistic regression analyses were performed to compare short- and long-term offspring outcomes between mothers receiving ACS treatment and those who did not among pregnancies with HDP and without HDP. Of 21,014 neonates, 4,806 (22.9%) were born to mothers with HDP. ACS treatment was associated with significant decreases in short-term adverse outcomes in the both HDP and non-HDP groups, with similar reduced odds of neonatal death, respiratory distress syndrome, and intraventricular haemorrhage (IVH). However, ACS treatment did not significantly decrease severe IVH (aOR 0.76; 95% CI 0.51–1.13) and periventricular leukomalacia (1.14; 0.78–1.66) in the HDP group. In addition, ACS treatment in mothers without HDP significantly decreased cerebral palsy (aOR 0.70; 95% CI 0.58–084), developmental quotient scores <85 (0.79; 0.69–0.90), and composite adverse outcomes (0.85; 0.75–0.96) at 3 years of age, whereas ACS treatment in mothers with HDP did not significantly improve these outcomes (1.04; 0.69–1.57, 1.11; 0.88–1.39, 0.96; 0.75–1.22, respectively). ACS treatment was associated with significantly decreased major short-term morbidities and mortality among extremely and very preterm neonates of mothers with HDP, with ACS treatment having a decreased effect compared to that observed in neonates of mothers without HDP. Although ACS treatment has no additional effects on offspring outcomes at 3 years of age, our results did not suggest that ACS treatment should be withheld from mothers with HDP.

## Introduction

Growing evidence suggests that the administration of antenatal corticosteroids (ACS) to women at risk of imminent preterm birth at <34 weeks of gestational age reduces neonatal morbidity and mortality^1–3^. ACS treatment has been estimated to decrease perinatal death by 28%, respiratory distress syndrome (RDS) by 34%, and intraventricular haemorrhage (IVH) by 45%, without increasing the risk of maternal sepsis and chorioamnionitis^1^. ACS treatment has become the standard of care. However, whether ACS treatment has similar beneficial effects on women with multiple pregnancies, fetal growth restriction, chorioamnionitis, and hypertensive disorders of pregnancy (HDP) has not been fully evaluated^4,5^.

HDP is a major pregnancy complication, to which approximately 20% of preterm births can be attributed^6^. In cases of HDP, especially preeclampsia, fetuses are exposed to chronic hypoxia, undernutrition, oxidative stress, various inflammatory cytokines, and anti-angiogenic factors^7^. This hostile environment can cause intrauterine stress, meaning that fetuses may already be exposed to elevated steroid levels even before receiving ACS treatment^8^. Thus, sufficient effect of ACS treatment on neonatal outcomes cannot be obtained in pregnancies complicated by HDP, contrary to our expectations. In addition, approximately 30–50% of women with early-onset HDP also have fetal growth restriction^9^, and data on the efficacy of ACS treatment in women with growth-restricted fetuses are limited and conflicting^4,5,10–12^. Thus, whether ACS treatment could decrease neonatal adverse outcomes in singleton pregnancies complicated by HDP similar to those observed in pregnancies without HDP remains an important unanswered question in clinical practice.

A previous meta-analysis demonstrated that ACS treatment was associated with reduced risk of cerebral palsy (CP) and severe disability at one year of age or later in children born preterm^13^. However, limited data on the long-term effect of ACS treatment among neonates born to mothers with HDP are available.

Thus, this study aimed to investigate the association between ACS treatment and short- and long-term outcomes of extremely and very preterm neonates born to mothers with HDP and of neonates born to mothers without HDP using a large nationwide database.

## Methods

### Study population

This population-based retrospective study was conducted based on an analysis of data collected by the Neonatal Research Network of Japan (NRNJ) between January 2003 and December 2016. The NRNJ database prospectively registered the clinical information on neonates born at 22–31 weeks of gestation and weighing ≤1,500 g admitted to the neonatal intensive care units (NICUs). More than 200 facilities accounting for 50–60% of level II and III NICUs in Japan participate in the NRNJ, and approximately 4,000 infants are registered in the database each year^14^. Designated clinical information on maternal and neonatal characteristics, pregnancy complications, and short- and long-term offspring outcomes was routinely collected by systematic medical record reviews and sent to the NRNJ Database Centre on a yearly basis. Patient information was anonymised and de-identified prior to analysis in the Database Centre. Informed consent was obtained from all patients at each facility to use the data for future neonatal care research. This study was approved by the institutional ethics boards of Nagoya University (approval number: 2018–0026) and the Japan Neonatal Network Executive Committee. Patients were not involved in data analysis and data interpretation.

Information on 48,569 neonates born weighing ≤1,500 g at 22–31 weeks of gestation that had been registered in the NRNJ database from 2003 to 2016 was obtained. Exclusion criteria for this study were defined as follows: less than 24 weeks of gestation, multiple pregnancies, major congenital abnormalities, out-of-hospital birth, and incomplete medical records including maternal characteristics.

### ACS treatment

According to the guidelines for obstetrical practice in Japan^15^, administration of ACS (injection of 12 mg of betamethasone intramuscularly followed by a repeat injection 24 hours later) is recommended for women at a risk of preterm birth within one week at less than 34 weeks of gestational age. Although information on the type of ACS, dose of ACS, and administration-to-birth interval in each case was not included in the NRNJ database, the majority of women in this study would have received a single course of betamethasone according to the guidelines, because only a single course of betamethasone was covered as ACS treatment by Japanese health insurance. Patients who received only one ACS injection until parturition were allocated to the ACS group.

### Definition

HDP was defined as hypertension (systolic blood pressure ≥140 mmHg and/or diastolic blood pressure ≥90 mmHg) during pregnancy^16^. The type of HDP, such as preeclampsia, gestational hypertension, superimposed preeclampsia, and chronic hypertension, was not documented in the NRNJ database.

Small for gestational age (SGA) was defined as both birth weight and height less than the 10^th^ percentile for gestational age based on a Japanese gender-specific neonatal anthropometric chart in 2000^17^. Diagnosis of respiratory distress syndrome (RDS) was based on a combination of clinical signs and chest radiography. Chronic lung disease (CLD) was defined by oxygen requirement at 36 weeks of postmenstrual age. Severe brain injury was defined as grade III or IV intraventricular haemorrhage (IVH) according to the classification of Papile et al. and periventricular leukomalacia (PVL) diagnosed by intracranial ultrasound or magnetic resonance imaging^18^. Neonatal sepsis was defined as clinical syndromes of bacteraemia with the presence of a pathogenic bacterium from a blood culture during NICU stay. Severe necrotizing enterocolitis (NEC) was diagnosed based on stage 2 or higher of Bell’s criteria^19^.

Neurodevelopmental outcomes of preterm infants were assessed using the Kyoto Scale of Psychological Development (KSPD) at 36 months by experienced clinical assessors at each facility. The KSPD is widely used and accepted as a developmental assessment tool in Japan^20,21^. The KSPD was reported to be comparable with the Bayley Scales of Infant and Toddler Development, Third edition (Bayley III)^20^. Therefore, the developmental quotient (DQ) assessed by KSPD is strongly correlated with the Bayley III scale^20^.

### Outcomes

The short-term offspring outcome measures were as follows: (1) in-hospital death; (2) respiratory morbidity, including RDS and CLD; (3) neurological injury, including IVH, and PVL; (4) NEC; and (5) neonatal sepsis. The short-term composite adverse outcomes were defined as one or more of the following: in-hospital death, severe IVH (grade III/IV), or PVL. In this study, short-term composite adverse outcomes included complications associated with death or severe brain injury.

The long-term offspring outcome measures were as follows: death after NICU discharge, home oxygen therapy/home respiratory therapy, visual impairment, hearing impairment, CP, and DQ < 70 at 3 years of age. The long-term composite adverse outcomes were defined as one of more of the following: death after NICU discharge, CP, and DQ < 70. Visual impairment was defined as blindness with no functional vision in at least one eye or bilateral amblyopia. Hearing impairment was defined as the need for hearing aids. In this study, long-term composite adverse outcomes included complications associated with death or neurodevelopmental impairment.

### Statistical analysis

Demographic and clinical characteristics were compared using the Chi-Square test for categorical variables and Student’s *t*-test for continuous variables. Univariate and multivariate logistic regression analyses were performed for the binary outcomes (e.g., in-hospital death, RDS, CLD, IVH, PVL, sepsis, NEC, short-term composite adverse outcomes, death after NICU discharge, home oxygen therapy/home respiratory therapy, visual impairment, hearing impairment, CP, DQ < 70, DQ < 85, and long-term composite adverse outcomes) to estimate the odds ratios (ORs) and 95% confidence intervals (CIs) for the prognostic variables. The multivariate logistic model included maternal age, parity, gestational age, mode of delivery, maternal gestational diabetes mellitus (GDM), diabetes mellitus (DM), premature rupture of membrane (PROM), histological chorioamnionitis (CAM), non-reassuring fetal status (NRFS), SGA, birth weight, and infant sex. To investigate whether the efficacy of ACS treatment varied across different groups (HDP vs non-HDP, HDP-SGA vs HDP-non-SGA), we also assessed the interaction between the group and the treatment (i.e., ACS) in the multivariate models. All the analyses performed in this study were exploratory: therefore, we did not adjust for multiplicity of testing. We considered a *p*-value of <0.05 to be significant. Statistical analysis was performed using SAS version 9.4 (SAS Institute Inc., Cary, NC, USA).

## Results

**Figure 1:**
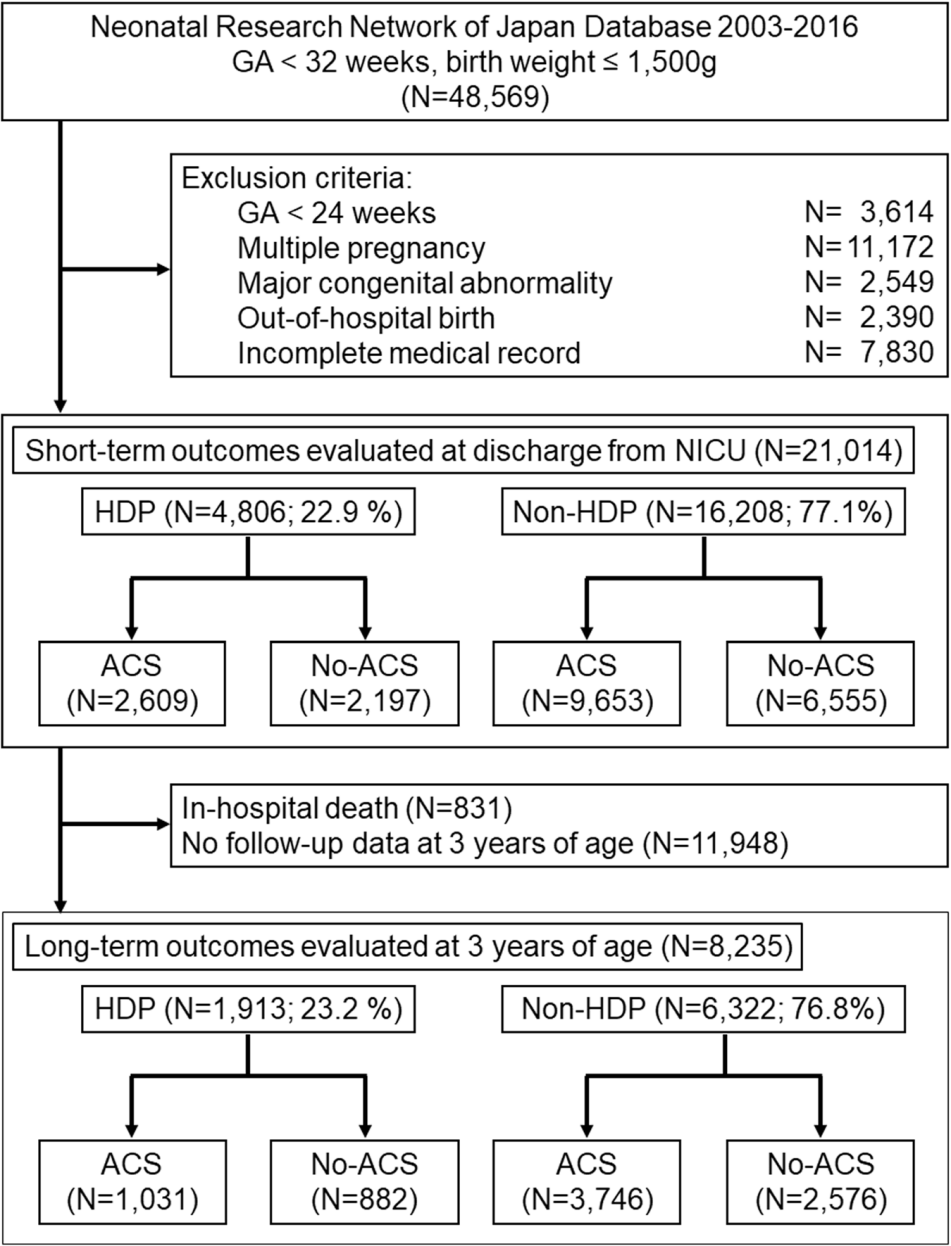
Flow diagram of the number of study patients. Data on 48,569 neonates registered in the NRNJ database from 2003 to 2016 were available. Short-term outcomes were assessed at discharge from the NICU, and long-term outcomes were assessed at 3 years of age. GA, gestational age; HDP, hypertensive disorders of pregnancy; ACS, antenatal corticosteroids. This figure was created using PowerPoint version 2013 (Microsoft Corporation, Washington, USA, https://products.office.com/en-us/powerpoint).

A total of 48,569 neonates born at the NICUs participating in the NRNJ were registered in the database during the study period. The final sample consisted of 21,014 singleton neonates born at 24–31 weeks of gestation whose short-term offspring outcomes were assessed at discharge from the NICU (HDP group; n = 4,806 [22.9%], non-HDP group; n = 16,208 [77.1%]) (Fig. 1). Of the 4,806 neonates in the HDP group, 2,609 (54.3%) received ACS treatment before birth, and 2,197 (45.7%) did not. Of the 16,208 neonates in the non-HDP group, 9,653 (59.6%) received ACS treatment, and 6,555 (40.4%) did not. Among the 21,014 neonates assessed for short-term outcomes, physical and developmental assessment at 3 years of age was performed in a total of 8,235 cases (HDP: n = 1,913 [23.2%]; non-HDP: n = 6,322 [76.8%]) (Fig. 1).

The baseline characteristics of the study groups are shown in Table 1. In both groups, mothers who received ACS treatment delivered at a slightly younger gestational age and had increased incidences of PROM and histological CAM than mothers who did not receive ACS treatment. In the non-HDP group, mothers who received ACS treatment were slightly older, primiparous, more likely to have GDM or DM, and less likely to show non-reassuring fetal status (NRFS) on cardiotocography compared with mothers who did not receive ACS treatment. The neonates born to mothers who received ACS treatment in the HDP group had lower birth weights and a significant increase in SGA. The neonates born to mothers who received ACS treatment in the non-HDP group had lower birth weights and heights, though the rate of SGA was not increased.

**Table 1:** Demographic and obstetric characteristics of the study population.

	HDP			Non-HDP		
	ACS	No-ACS	*p*-value	ACS	No-ACS	*p*-value
	(N = 2,609)	(N = 2,197)		(N = 9,653)	(N = 6,555)	
Maternal characteristics						
Maternal age (year)	33.9 ± 5.0	33.7 ± 5.1	0.19	31.4 ± 5.3	31.0 ± 5.5	<0.01
Primiparous (%)	1,519 (58.2)	1,235 (56.2)	0.16	4,649 (48.2)	3,051 (46.5)	0.04
Gestational age (wks)	28.9 ± 2.1	29.0 ± 2.0	0.01	27.7 ± 2.1	27.9 ± 2.1	<0.01
CS ratio (%)	2,505 (96.0)	2,127 (96.8)	0.14	6,846 (70.9)	4,748 (72.4)	0.04
GDM/DM (%)	123 (4.7)	90 (4.1)	0.30	318 (3.2)	176 (2.7)	0.03
Histological CAM (%)	413 (15.8)	258 (11.7)	<0.01	5,094 (52.8)	2,778 (42.4)	<0.01
PROM (%)	117 (4.5)	70 (3.2)	0.02	5,018 (52.0)	2,301 (35.1)	<0.01
NRFS (%)	967 (37.1)	842 (38.3)	0.37	2,415 (25.0)	1,898 (29.0)	<0.01
Neonatal characteristics						
Male (%)	1,212 (46.5)	1,021 (46.5)	0.99	5,175 (53.6)	3,522 (53.7)	0.88
Birth weight (g)	902 ± 282	952 ± 287	<0.01	998 ± 277	1,027 ± 279	<0.01
Height (cm)	33.9 ± 3.9	34.6 ± 3.9	<0.01	34.8 ± 3.6	35.2 ± 3.6	<0.01
Head circumference (cm)	25.0 ± 2.6	25.4 ± 2.7	<0.01	25.0 ± 2.5	25.3 ± 2.5	<0.01
SGA (%)	1,511 (57.9)	1,088 (49.5)	<0.01	1,265 (13.1)	838 (12.8)	0.55

CS; caesarean section, GDM; gestational diabetes mellitus, DM; diabetes mellitus, CAM; chorioamnionitis, PROM; premature rupture of membrane, NRFS; non-reassuring fetal status, SGA; small for gestational age, HDP; hypertensive disorders of pregnancy, ACS; antenatal corticosteroids. Data are presented as mean ± standard deviation or n (%).

Neonates born to mothers receiving ACS in both the HDP and non-HDP groups had lower rates of mortality and RDS, but higher rate of CLD compared with neonates born to mothers not receiving ACS (Table 2). ACS treatment in the HDP group did not significantly decrease the rates of severe IVH (grade III/IV), PVL, sepsis, and short-term composite adverse outcomes, which was in contrast to the non-HDP group. With respect to long-term outcomes, ACS treatment was associated with a significant decrease in CP, DQ < 85, and composite adverse outcomes at 3 years of age in the non-HDP group. Conversely, in the HDP group, no significant differences were observed with respect to the rates of mortality, severe disabilities, CP, and neurodevelopmental delay between the offspring with and without ACS treatment. Supplementary Tables 1 and 2 list the baseline characteristics and outcomes in offspring with and without a 3-year follow-up after excluding offspring who died by the age of three. In the non-HDP group, short-term outcomes without follow-up tended to be worse compared to those with 3-year follow-up.

**Table 2:** Short- and long-term offspring outcomes in the HDP and non-HDP groups - univariate analysis.

	HDP			Non-HDP		
	ACS	No-ACS	Crude OR (95% CI)	ACS	No-ACS	Crude OR (95% CI)
Short-term outcomes	(N = 2,609)	(N = 2,197)		(N = 9,653)	(N = 6,555)	
In-hospital death (%)	68/2,608 (2.6)	81/2,196 (3.7)	**0.70 (0.50–0.97)**	322/9,646 (3.3)	360/6,555 (5.5)	**0.59 (0.51–0.69)**
Respiratory distress syndrome (%)	1,749/2,608 (67.1)	1,543/2,196 (70.3)	**0.86 (0.76–0.97)**	5,757/9,633 (59.8)	4,426/6,548 (67.6)	**0.71 (0.67–0.76)**
Chronic lung disease (%)	673/2,586 (26.0)	428/2,178 (19.7)	**1.44 (1.25–1.65)**	2,682/9,566 (28.0)	1,338/6,493 (20.6)	**1.50 (1.39–1.62)**
Intraventricular haemorrhage (III or IV) (%)	51/2,597 (2.0)	55/2,189 (2.5)	0.78 (0.53–1.14)	334/9,603 (3.5)	418/6,511 (6.4)	**0.53 (0.45–0.61)**
Periventricular leukomalacia (%)	67/2,602 (2.6)	48/2,190 (2.2)	1.18 (0.81–1.72)	293/9,620 (3.0)	336/6,529 (5.1)	**0.58 (0.49–0.68)**
Sepsis (%)	160/2,603 (6.1)	152/2,188 (6.9)	0.88 (0.70–1.10)	782/9,631 (8.1)	607/6,538 (9.3)	**0.86 (0.77–0.96)**
Necrotizing enterocolitis (%)	28/2,608 (1.1)	34/2,194 (1.5)	0.69 (0.42–1.14)	149/9,632 (1.5)	101/6,545 (1.5)	1.00 (0.78–1.29)
Composite adverse outcomes (%)	162/2,609 (6.2)	159/2,197 (7.2)	0.85 (0.68–1.06)	826/9,653 (8.6)	931/6,555 (14.2)	**0.57 (0.51–0.62)**
Long-term outcomes	(N = 1,031)	(N = 882)		(N = 3,746)	(N = 2,576)	
Death after NICU discharge (%)	8/1,031 (0.8)	4/882 (0.5)	1.72 (0.52–5.72)	37/3,746 (1.0)	28/2,576 (1.1)	0.91 (0.55–1.49)
Home oxygen therapy/home respiratory therapy (%)	14/892 (1.6)	14/731 (1.9)	0.82 (0.39–1.70)	68/3,111 (2.2)	36/2,024 (1.8)	1.23 (0.82–1.86)
Visually impairment (%)	47/942 (5.0)	36/827 (4.4)	1.15 (0.74–1.80)	1,91/3,402 (5.6)	142/2,347 (6.1)	0.92 (0.74–1.16)
Hearing impairment (%)	4/713 (0.6)	6/631 (1.0)	0.61 (0.18–2.04)	42/2,725 (1.5)	38/1,793 (2.1)	0.72 (0.46–1.13)
Cerebral palsy (%)	58/975 (5.9)	46/851 (5.4)	1.11 (0.74–1.65)	271/3,510 (7.7)	259/2,458 (10.5)	**0.71 (0.59–0.85)**
Developmental quotient <70 (%)	117/743 (15.7)	93/593 (15.7)	1.00 (0.75–1.35)	416/2,612 (15.9)	274/1,643 (16.7)	0.95 (0.80–1.12)
Developmental quotient <85 (%)	364/743 (49.0)	268/593 (45.2)	1.16 (0.94–1.45)	1,283/2,612 (49.1)	874/1,643 (53.2)	**0.85 (0.75–0.96)**
Composite adverse outcomes (%)	158/1,031 (15.3)	131/882 (14.9)	1.02 (0.80–1.29)	648/3,746 (17.3)	494/2,576 (19.2)	**0.88 (0.78–0.99)**

HDP, hypertensive disorders of pregnancy; ACS, antenatal corticosteroids; OR, odds ratio; Short-term composite adverse outcomes: in-hospital death, intraventricular haemorrhage (grade III or IV) and periventricular leukomalacia, Long-term composite adverse outcomes: death after NICU discharge, cerebral palsy and developmental quotient <70. Bold indicates a significant association.

**Figure 2:**
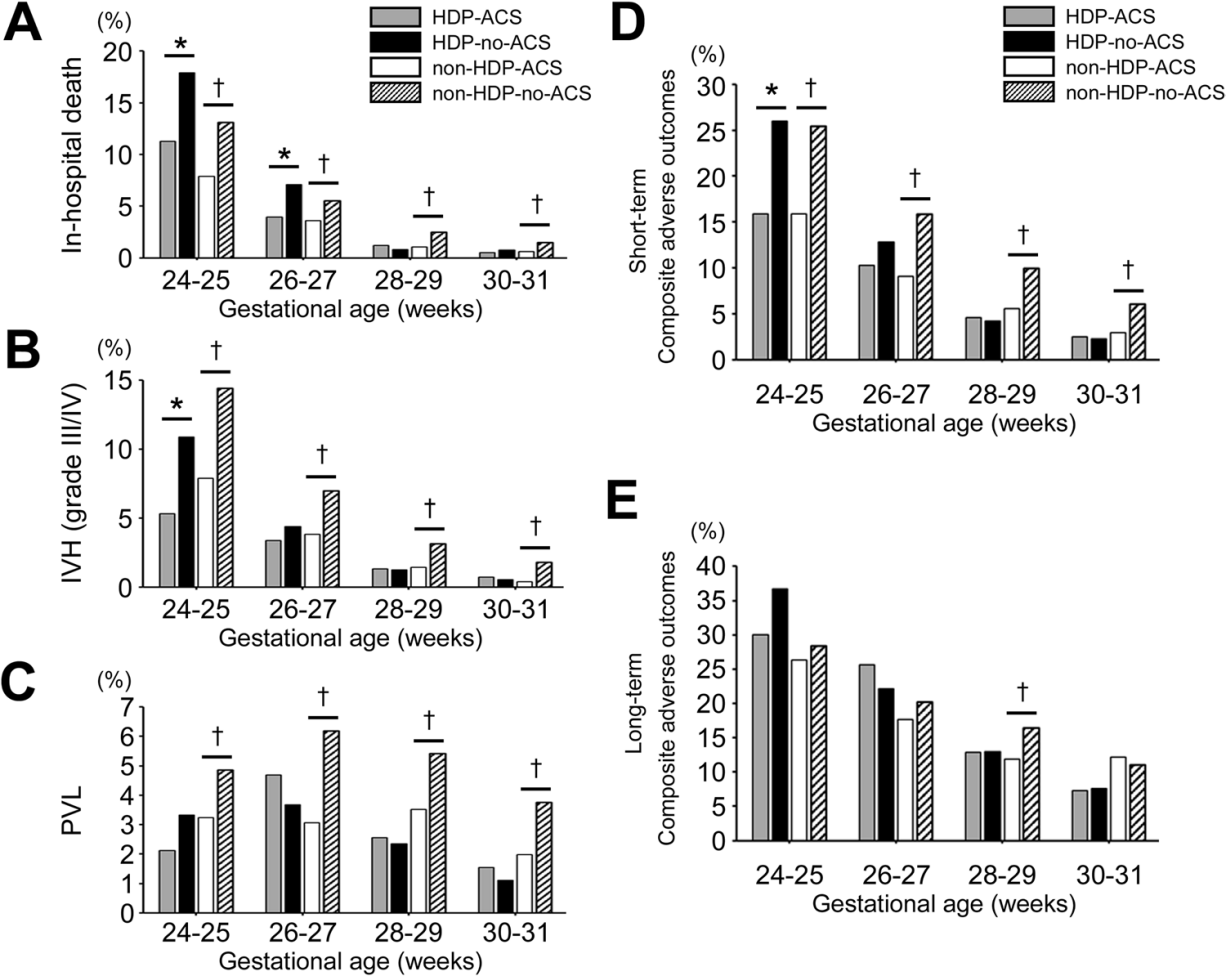
Short- and long-term offspring outcomes by gestational week at birth according to exposure to ACS. The rates of (**A**) in-hospital death, (**B**) IVH (grade III/IV), (**C**) PVL, (**D**) short-term composite adverse outcomes and (**E**) long-term composite adverse outcomes by gestational week at birth in the HDP and non-HDP groups according to exposure to ACS. *P < 0.05 for ACS treatment versus no ACS treatment in the HDP group. ^†^*P* < 0.05 for ACS treatment versus no ACS treatment in the non-HDP group. IVH, intraventricular haemorrhage; PVL, periventricular leukomalacia. Short-term composite adverse outcomes: in-hospital death, severe IVH (grade III/IV), and PVL. Long-term composite adverse outcomes: death after NICU discharge, cerebral palsy, and developmental quotient <70.

Figure 2 shows the rates of short- and long-term composite adverse offspring outcomes by gestational week at birth in the HDP and non-HDP groups according to exposure to ACS. When stratified by gestational week at birth, short- and long-term outcomes decreased with each successive week, by which a decreased effect of ACS was observed on short-term outcomes in the HDP group compared with the non-HDP group. However, the prevalence of IVH (grade III/IV), PVL, and short-term composite adverse outcomes in the HDP group without ACS was lower than that in the non-HDP group without ACS at all gestational weeks. The effect of ACS treatment on long-term outcomes was comparable between the two groups, except for at 28–29 weeks of gestation in the non-HDP group.

A multivariate logistic regression analysis was performed to determine the efficacy of ACS treatment on neonatal outcomes in the HDP and non-HDP groups (Table 3). This analysis was adjusted for maternal age, parity, gestational age, mode of delivery, GDM/DM, PROM, histological CAM, NRFS, SGA, birth weight, and infant sex. Neonates born to mothers not receiving ACS were used as a reference in both groups. Regarding short-term outcomes, the effect of ACS treatment in mothers with HDP was similar to that observed in the non-HDP group; however, ACS treatment was not associated with significant decreases in severe IVH, PVL, and sepsis in the HDP group contrary to the non-HDP group (*p*-value for interaction = 0.08, <0.01, and 0.93, respectively). Furthermore, ACS treatment did not improve long-term outcomes in the HDP group. A *p-*value for interaction <0.05 indicates a significant difference in the effect of ACS treatment between the two groups. Therefore, ACS treatment in the HDP group showed significantly decreased protective effects against the development of PVL, short-term composite adverse outcomes, CP, and DQ < 85 at 3 years of age compared with the non-HDP group.

**Table 3:** Association between ACS treatment and offspring adverse outcomes among neonates born to mothers with and without ACS treatment by multivariate analysis.

	HDP:ACS vs No-ACS	Non-HDP: ACS vs No-ACS	interaction
	aOR (95% CI)	aOR (95% CI)	*p*-value
Short-term outcomes			
In-hospital death	**0.56 (0.39–0.79)**	**0.56 (0.47–0.65)**	0.63
Respiratory distress syndrome	**0.85 (0.74–0.97)**	**0.77 (0.72–0.83)**	0.09
Chronic lung disease	**1.35 (1.15–1.58)**	**1.39 (1.28–1.51)**	0.52
Intraventricular haemorrhage (III or IV)	0.76 (0.51–1.13)	**0.52 (0.44–0.60)**	0.08
Periventricular leukomalacia	1.14 (0.78–1.66)	**0.58 (0.49–0.69)**	<0.01
Sepsis	0.79 (0.62–1.01)	**0.81 (0.72–0.91)**	0.93
Necrotizing enterocolitis	0.64 (0.39–1.04)	0.94 (0.72–1.22)	0.13
Composite adverse outcomes	**0.75 (0.60–0.96)**	**0.55 (0.49–0.61)**	<0.01
Long-term outcomes			
Death after NICU discharge	1.84 (0.54–6.33)	0.88 (0.53–1.46)	0.39
Home oxygen therapy/home respiratory therapy	0.74 (0.37–1.48)	1.01 (0.66–1.53)	0.47
Visually impairment	1.10 (0.69–1.73)	0.86 (0.68–1.08)	0.34
Hearing impairment	0.54 (0.19–1.51)	0.77 (0.49–1.21)	0.65
Cerebral palsy	1.04 (0.69–1.57)	**0.70 (0.58–0.84)**	0.03
Developmental quotient <70	0.91 (0.66–1.25)	0.90 (0.75–1.07)	0.89
Developmental quotient <85	1.11 (0.88–1.39)	**0.79 (0.69–0.90)**	0.03
Composite adverse outcomes	0.96 (0.75–1.22)	**0.85 (0.75–0.96)**	0.39

Model adjusted for variables including maternal age, parity, gestational age, mode of delivery, GDM/DM, PROM, histological CAM, NRFS, SGA, birth weight and infant sex. Neonates born to mothers without ACS treatment were used as a reference in both groups. HDP, hypertensive disorders of pregnancy; ACS, antenatal corticosteroids; aOR, adjusted odds ratio; Short-term composite adverse outcomes: in-hospital death, intraventricular haemorrhage (grade III or IV) and periventricular leukomalacia, Long-term composite adverse outcomes: death after NICU discharge, cerebral palsy and developmental quotient <70. Bold indicates a significant association. *P-*value for interaction <0.05 indicates a significant difference in the effect of ACS treatment between the two groups.

To identify the reason for the decreased effect of ACS treatment in women with HDP, we performed a subgroup analysis stratified by the presence of SGA. Comparing the effect of ACS treatment between the cases of HDP with and without SGA, the effects of ACS treatment were slightly different. However, the presence of SGA in the HDP group did not significantly alter the effect of ACS treatment on short- and long-term offspring outcomes (Table 4).

**Table 4:** Subgroup multivariate analysis of the association between ACS treatment and adverse offspring outcomes between the HDP-SGA and HDP-non-SGA groups.

	HDP-SGA:	HDP-non-SGA:	
	ACS vs No-ACS	ACS vs No-ACS	interaction
	aOR (95% CI)	aOR (95% CI)	*p*-value
Short-term outcomes			
In-hospital death	**0.48 (0.31–0.73)**	0.80 (0.43–1.50)	0.43
Respiratory distress syndrome	0.89 (0.74–1.07)	**0.79 (0.64–0.97)**	0.39
Chronic lung disease	**1.37 (1.12–1.68)**	**1.33 (1.04–1.69)**	0.93
Intraventricular haemorrhage (III or IV)	0.81 (0.48–1.37)	0.71 (0.39–1.27)	0.54
Periventricular leukomalacia	1.06 (0.63–1.76)	1.18 (0.69–2.05)	0.88
Sepsis	**0.68 (0.50–0.91)**	1.06 (0.71–1.57)	0.08
Necrotizing enterocolitis	0.67 (0.37–1.24)	0.55 (0.25–1.19)	0.73
Composite adverse outcomes	**0.67 (0.49–0.91)**	0.90 (0.62–1.30)	0.49
Long-term outcomes			
Death after NICU discharge	1.30 (0.38–4.44)	1.97 (0.50–7.78)	0.99
Home oxygen therapy/home respiratory therapy	1.29 (0.49–3.43)	0.43 (0.14–1.29)	0.1
Visually impairment	0.91 (0.50–1.67)	1.60 (0.81–3.17)	0.33
Hearing impairment	0.33 (0.09–1.18)	2.00 (0.46–8.72)	0.22
Cerebral palsy	1.15 (0.65–2.04)	1.08 (0.58–2.01)	0.73
Developmental quotient <70	0.76 (0.50–1.14)	1.25 (0.75–2.10)	0.15
Developmental quotient <85	1.17 (0.86–1.60)	1.11 (0.79–1.57)	0.62
Composite adverse outcomes	0.85 (0.62–1.17)	1.18 (0.80–1.74)	0.3

Model adjusted for variables including maternal age, parity, gestational age, mode of delivery, GDM/DM, PROM, histological CAM, NRFS, birth weight and infant sex. Neonates born to mothers without ACS treatment were used as a reference in both groups. SGA, small for gestational age; HDP, hypertensive disorders of pregnancy; ACS, antenatal corticosteroids; aOR, adjusted odds ratio; Short-term composite adverse outcomes: in-hospital death, intraventricular haemorrhage (grade III or IV) and periventricular leukomalacia, Long-term composite adverse outcomes: death after NICU discharge, cerebral palsy and developmental quotient <70. Bold indicates a significant association. *P*-value for interaction <0.05 indicates a significant difference in the effect of ACS treatment between the two groups.

## Discussion

In this population-based study, ACS treatment to mothers with HDP was associated with significantly decreased major short-term morbidities and mortality among extremely and very preterm neonates, with ACS treatment having a slightly decreased effect compared with that observed in neonates born to mothers without HDP. ACS treatment was not found to exert a beneficial effect on long-term offspring outcomes, including cerebral palsy and neurodevelopmental delay at 3 years of age in the HDP group. We also demonstrated that the presence of SGA in the HDP group did not significantly alter the effect of ACS treatment on short- and long-term offspring outcomes.

Overall, the effect of ACS treatment on short-term neonatal outcomes in the HDP group was similar to that in the non-HDP group, in line with previous studies demonstrating decreased risk of mortality, RDS, and IVH^1,22^. In addition, a previous meta-analysis addressed the efficacy of ACS treatment on offspring neurodevelopmental outcomes at one year of age or later and showed a reduced risk of CP, severe disability, and psychomotor developmental index <70^13^. The long-term effect of ACS treatment in the non-HDP group was consistent with that reported in previous study^13^. However, a significant decrease in severe brain injury at short-term and neuroprotective effect of ACS treatment at 3 years of age were not observed in the HDP group.

The limited effect of ACS treatment on preterm short- and long-term offspring outcomes in the HDP group must be interpreted with caution. Possible explanations for this include the following: (1) the prevalence of neonatal morbidities in the HDP group may already be low; (2) the level of glucocorticoids may be higher due to endogenous production of fetal glucocorticoids before ACS treatment; (3) more than half of the preterm neonates in the HDP group exhibited SGA, which was reported to be decreased effect of ACS treatment; and (4) several perinatal factors (e.g. administration-to-birth interval of ACS and aetiology of preterm birth) may be associated with the efficacy of ACS treatment and long-term offspring outcomes.

Firstly, the prevalence of IVH, PVL and short-term composite adverse outcomes in the HDP group without ACS was lower than those in the non-HDP group without ACS at all gestational weeks. This indicated that obtaining a significant difference regarding ACS efficacy was difficult due to the lower rate of neonatal morbidities in the HDP group. In contrast, the higher rate of neonatal morbidities in the non-HDP group might be attributed to chorioamnionitis, which is known to have a negative impact on subsequent neurological and physical development^23^.

Secondly, in the HDP cases, especially preeclampsia, fetuses are exposed to chronic intrauterine stress, such as hypoxia, oxidative stress, various cytokines, and anti-angiogenic factors. This intrauterine stress has been implicated in the disruption of the hypothalamic–pituitary–adrenal (HPA) axis, leading to increased levels of fetal cortisol^24,25^. Therefore, endogenous production of fetal glucocorticoids may be increased before exposure to ACS, which may help to explain the absence of an additional effect from ACS treatment^26,27^, as well as the decreased prevalence of neonatal morbidity, even in cases without ACS treatment. This hypothesis is supported by a study that showed that the cortisol level of umbilical cord blood was significantly increased in fetuses in pregnancies with preeclampsia^8^.

Thirdly, previous reports showed a slightly decreased effect of ACS treatment on neonatal outcomes, such as RDS and IVH, in cases of SGA^4,10^. In our study, more than half of the preterm neonates in the HDP group exhibited SGA. Statistically, the presence of SGA in the HDP group did not significantly alter the effect of ACS treatment on short- and long-term offspring outcomes (interaction *p* ≥ 0.05); however, the effect of ACS treatment was considerably different.

Finally, perinatal factors such as administration-to-birth interval of ACS, repeated course of ACS and aetiology of preterm birth may have differed among the patients, and these factors may have affected the neonatal outcomes^28,29^. A previous study demonstrated that a reduction of severe neonatal brain injury by ACS treatment largely varied according to the administration-to-birth interval of ACS^29^. Thus, there is a possibility that patients in the HDP group were likely to receive an incomplete ACS treatment (e.g. receive only one injection or receive two injections, but deliver immediately) or deliver after 7 days of ACS treatment. However, the NRNJ database did not include these data.

It is also important to note that ACS treatment might have a potentially harmful effect on neonates if given unnecessarily (e.g. women suspected of preterm labour who do not actually deliver at preterm). In this study, ACS treatment increased CLD occurrence in both the HDP and non-HDP groups, even though ACS treatment decreased RDS occurrence in both groups. Consistent with our study, the previous report that used the same database, but for a different period, showed an increased incidence of CLD following ACS treatment^30^. A definitive explanation for this is impossible without further research; however, one possible explanation is that the improved survival rate attributable to ACS treatment might increase the number of neonates requiring long-term ventilation. Thus, we speculated that the increased incidence of CLD secondary to ACS treatment did not necessarily indicate a harmful event. Additionally, the negative effects of ACS treatment on maternal health should be addressed in future studies.

The first strength of this study is that it is the first to report on short- and long-term effects of ACS treatment on preterm neonates born to mothers with HDP. Second, we obtained a sample size larger than those used in previous reports by accessing a nationwide database^22^.

Several limitations of this study should be acknowledged. First, the NRNJ database did not include certain maternal information such as the type and severity of HDP, dose and administration-to-birth interval of ACS, and detailed characteristics such as the use of magnesium sulphate, pre-pregnancy BMI, smoking history, etc. In addition, the causes of preterm delivery (iatrogenic or spontaneous) and the reason why the mothers could not receive ACS treatment (e.g. placental abruption, non-reassuring fetal status, and eclampsia) were not available. A recent study on a national inpatient database in Japan showed that the administration-to-birth interval of ACS was 3 (1–9) days (median-interquartile ranges), an appropriate time to elicit maximum effectiveness; however, we could not compare it between the two groups in this database^31^. Second, among the 21,014 infants discharged from NICUs, approximately 60% of the infants registered in the NRNJ were lost to follow-up at 3 years of age. Thus, we cannot exclude the possibility of selection bias in long-term offspring outcomes. Although we could not find any additional effects from ACS treatment in the HDP group on offspring neurodevelopmental disorders at 3 years of age, including CP and neurodevelopmental delay, our results on the long-term effects need be interpreted with caution. Further research will be required to investigate the ACS effects in different populations (e.g. different gestational age, race, and region). Finally, because this study was not a multi-institutional joint research study but rather a multicentre retrospective study, it should be considered that there are different strategies for preterm birth or HDP at different facilities, though those differences were likely minimized by the large size of the study population.

## Conclusion

We demonstrated that administration of ACS to women with HDP resulted in a beneficial effect on major short-term neonatal outcomes. This benefit was similar in magnitude to the benefit observed among mothers without HDP. We could not find any additional effects of ACS treatment in the HDP group on offspring outcomes such as severe brain injury in the short-term or neurodevelopmental disorders at 3 years of age, including CP and neurodevelopmental delay. However, these results must be interpreted with caution; our results did not suggest that ACS treatment should be withheld from mothers with HDP. In addition, further investigation is required to verify our findings in different populations.

## Supplementary information

Supplementary information.

## Data Availability

The data that support the findings of this study are available from Neonatal Research Network of Japan but restrictions apply to the availability of these data, which were used under license for the current study, and so are not publicly available. Data are however available from the authors upon reasonable request and with permission of Neonatal Research Network of Japan.
