# Supplementary material for: Antenatal corticosteroids and preterm offspring outcomes in hypertensive disorders of pregnancy: A Japanese cohort study
Source: Sci Rep. 2020 Jun 9;10:9312. doi: 10.1038/s41598-020-66242-z (PMC7283214; doi:10.1038/s41598-020-66242-z)
Supplement: Supplementary file 1 — Supplementary information. [file 41598_2020_66242_MOESM1_ESM.pdf]

Antenatal corticosteroids and preterm offspring outcomes in hypertensive disorders of pregnancy: A Japanese cohort study

Takafumi Ushida[1], Tomomi Kotani[1,*], Masahiro Hayakawa[2,4], Akihiro Hirakawa[3], Ryo Sadachi[3], Noriyuki Nakamura[1], Yoshinori Moriyama[1], Kenji Imai[1], Tomoko Nakano-Kobayashi[1], Fumitaka Kikkawa[1]

[1]Department of Obstetrics and Gynecology, Nagoya University Graduate School of Medicine, Nagoya, Japan

[2]Division of Neonatology, Center for Maternal-Neonatal Care, Nagoya University Hospital, Nagoya, Japan

[3]Department of Biostatistics and Bioinformatics, Graduate School of Medicine, The University of Tokyo, Tokyo, Japan

[4] Neonatal Research Network of Japan

Supplementary Table 1. Demographic and obstetric characteristics in the offspring with 3-year follow-up and without 3-year follow-up.

	HDP			Non-HDP		
	With follow-up (N=1,901)	Without follow-up (N=2,745)	*p*-value	With follow-up (N=6,256)	Without follow-up (N=9,211)	*p*-value
Maternal characteristics						
Maternal age (year)	33.6±5.0	34.0±5.1	0.01	31.3±5.2	31.2±5.5	0.55
Primiparous (%)	1,105 (58.1)	1,557 (56.7)	0.34	3,037 (48.5)	4,318 (46.8)	0.04
Gestational age (wks)	28.9±2.0	29.1±2.0	0.03	27.7±2.1	28.0±2.1	<0.01
CS ratio (%)	1,830 (96.3)	2,647 (96.4)	0.76	4,483 (71.7)	6,565 (71.3)	0.60
ACS (%)	1,023 (53.8)	1,510 (55.0)	0.42	3,708 (59.2)	5,590 (60.7)	0.08
GDM/DM (%)	78 (4.1)	128 (4.7)	0.36	174 (2.8)	309 (3.4)	0.04
Histological CAM (%)	232 (12.2)	411 (15.0)	<0.01	2,946 (47.1)	4,600 (49.9)	<0.01
PROM (%)	69 (3.6)	108 (3.9)	0.60	2,867 (45.8)	4,152 (45.1)	0.36
NRFS (%)	718 (37.8)	1,017 (37.1)	0.62	1,604 (25.6)	2,413 (26.2)	0.44
Neonatal characteristics						
Male (%)	871 (45.8)	1,283 (46.7)	0.54	3,387 (54.1)	4,888 (53.1)	0.19
Birth weight (g)	925±276	944±281	0.02	1,000±271	1,035±276	<0.01
Height (cm)	34.3±3.8	34.5±3.8	0.08	34.8±3.6	35.3±3.6	<0.01
Head circumference (cm)	25.2±2.6	25.3±2.6	0.04	25.1±2.5	25.3±2.4	<0.01
SGA (%)	1,024 (53.9)	1,464 (53.3)	0.72	836 (13.4)	1,094 (11.9)	<0.01

CS; caesarean section, ACS; antenatal corticosteroids, GDM; gestational diabetes mellitus, DM; diabetes mellitus, CAM; chorioamnionitis, PROM; premature rupture of membrane, NRFS; non-reassuring fetal status, SGA; small for gestational age, HDP; hypertensive disorders of pregnancy. Data are presented as mean ± standard deviation or n (%).

Supplementary Table 2. Short-term offspring outcomes in the offspring with 3-year follow-up and without 3-year follow-up.

	HDP			Non-HDP		
	With follow-up (N=1,901)	Without follow-up (N=2,745)	*p*-value	With follow-up (N=6,256)	Without follow-up (N=9,211)	*p*-value
Short-term outcomes	(N=1,901)	(N=2,745)		(N=6,256)	(N=9,211)	
Respiratory distress syndrome (%)	1,288/1,900 (67.8)	1,882/2,744 (68.6)	0.57	3,878/6,253 (62.0)	5,784/9,194 (62.9)	0.26
Chronic lung disease (%)	445/1,893 (23.5)	633/2,713 (23.3)	0.89	1,523/6,222 (24.5)	2,374/9,102 (26.1)	0.03
Intraventricular haemorrhage (III or IV) (%)	27/1,894 (1.4)	44/2,732 (1.6)	0.72	210/6,228 (3.4)	357/9,160 (3.9)	0.09
Periventricular leukomalacia (%)	47/1,896 (2.5)	63/2,737 (2.3)	0.70	204/6,247 (3.3)	391/9,176 (4.3)	<0.01
Sepsis (%)	112/1,896 (5.9)	146/2,735 (5.3)	0.41	463/6,246 (7.4)	670/9,187 (7.3)	0.78
Necrotizing enterocolitis (%)	12/1,900 (0.6)	24/2,742 (0.9)	0.35	63/6,254 (1.0)	109/9,188 (1.2)	0.30
Composite adverse outcomes (%)	70/1,901 (3.7)	101/2,745 (3.7)	1.00	375/6,256 (6.0)	688/9,211 (7.5)	<0.01

HDP, hypertensive disorders of pregnancy; Short-term composite adverse outcomes: intraventricular haemorrhage (grade III or IV) and periventricular leukomalacia.

Appendix

Institutions enrolled in the study of the Neonatal Research Network of Japan were as follows: Sapporo City General Hospital, Asahikawa-Kosei General Hospital, Engaru-Kosei General Hospital, Kushiro Red Cross Hospital, Obihiro-Kosei General Hospital, Tenshi Hospital, NTT East Sapporo Hospital, Nikko Memorial Hospital, Nayoro City General Hospital, Sapporo Medical University, Asahikawa Medical University, Aomori Prefectural Central Hospital, Iwate Medical University, Iwate Prefectural Ofunato Hospital, Iwate Prefectural Kuji Hospital, Iwate Prefectural Ninohe Hospital, Sendai Red Cross Hospital, Tohoku University, Akita Red Cross Hospital, Akita University, Tsuruoka Municipal Shonai Hospital, Yamagata University, Yamagata Prefectural Central Hospital, Fukushima Medical University, Takeda General Hospital, Fukushima National Hospital, Tsukuba University, Tsuchiura Kyodo General Hospital, Ibaraki Children's Hospital, Dokkyo Medical University, Jichi Medical University, Ashikaga Red Cross Hospital, Gunma Children's Medical Center, Kiryu Kosei General Hospital, Ohta General Hospital, Gunma University, Saitama Medical University, Saitama Children's Medical Center, Nishisaitama-Chuo National Hospital, Saitama Medical University Saitama Medical Center, Kawaguchi Municipal Medical Center, Jichi Medical University Saitama Medical Center, Asahi General Hospital, Chiba Kaihin Municipal Hospital, Kameda Medical Center, Tokyo Women's Medical University Yachiyo Medical Center, Juntendo University Urayasu Hospital, Narita Red Cross Hospital, Tokyo Metropolitan Children's Medical Center, Tokyo Women's Medical University, Aiiku Hospital, Nihon University, National Center for Global Health and Medicine, Tokyo Medical University, Teikyo University, Showa University, Japan Red Cross Medical Center, National Center for Child Health and Development, Tokyo Metropolitan Ohtsuka Hospital, Tokyo University, Toho University, Tokyo Metropolitan Bokuto Hospital, Tokyo Jikei Medical University, Tokyo Medical and Dental University, St. Luke’s international Hospital, Juntendo University, Sanikukai Hospital, Katsushika Red Cross Maternity Hospital, Yokohama Rosai Hospital, Yokohama City University Medical Center, St. Marianna University School of Medicine, Kanagawa Children's Medical Center, Tokai University, Kitazato University, Odawara Municipal Hospital, Nippon Medical School Musashi Kosugi Hospital, Yokohama City Hospital, Saiseikai Yokohamashi Tobu Hospital, National Hospital Organization Yokohama Medical Center, Yamanashi Prefectural Central Hospital, Nagano Children's Hospital, Shinshu University, Iida Municipal Hospital, National Hospital Organization Shinshu Ueda Medical Center, Saku General Hospital, Nigata University, Niigata Prefectural Central Hospital, Niigata City General Hospital, Nagaoka Red Cross Hospital, Kouseiren Takaoka Hospital, Toyama Prefectural Central Hospital, Toyama University, Ishikawa Prefectural Central Hospital, Kanazawa Medical University, National Hospital Organization Kanazawa Medical Center, Fukui Prefectural Hospital, Fukui University, Gifu Prefectural General Medical Center, Ogaki Municipal Hospital, National Hospital Organization Nagara Medical Center, Takayama Red Cross

Hospital, Seirei Hamamatsu General Hospital, Shizuoka Saiseikai General Hospital, Shizuoka Children's Hospital, Hamamatsu University Hospital, Numazu City Hospital, Yaizu City Hospital, Fujieda Municipal General Hospital, Nagoya Red Cross Daini Hospital, Nagoya University, Nagoya Red Cross Daiichi Hospital, Toyohashi Municipal Hospital, Nagoya City West Medical Center, Fujita Health University, Anjo Kosei Hospital, Tosei General Hospital, Komaki City Hospital, Toyota Memorial Hospital, Okazaki City Hospital, Handa City Hospital, Konan Kosei Hospital, Aichi Medical University, National Hospital Organization Mie Chuo Medical Center, Ise Red Cross Hospital, Yokkaichi Municipal Hospital, Otsu Red Cross Hospital, Shiga Medical University, Nagahama Red Cross Hospital, Uji-Tokushukai Medical Center, The Japan Baptist Hospital, Kyoto University, Kyoto Red Cross Daiichi Hospital, National Hospital Organization Maizuru Medical Center, Fukuchiyama City Hospital, Kyoto Prefecture University of Medicine, Kyoto City Hospital, Mitsubishi Kyoto Hospital, Yodogawa Christian Hospital, Osaka Medical Center and Research Institute for Maternal and Child Health, Osaka University, Takatsuki General Hospital, Kansai Medical University, Osaka City General Hospital, Osaka City Sumiyoshi Hospital, Aizenbashi Hospital, Toyonaka Municipal Hospital, National Cerebral and Cardiovascular Center, Kitano Hospital, Saiseikai Suita Hospital, Chibune General Hospital, Bell Land General Hospital, Rinku General Medical Center, Osaka Red Cross Hospital, Yao Municipal Hospital, Hannan Chuo Hospital, Osaka General Medical Center, Osaka City University, Kobe Children's Hospital, Kobe University, Kakogawa City Hospital, Saiseikai Hyogo Hospital, Kobe City Medical Center General Hospital, Hyogo Medical University, Himeji Red Cross Hospital, Toyooka Hospital, Hyogo Prefectural Awaji Medical Center, Nara Medical University, Wakayama Medical University, Tottori Prefectural Central Hospital, Tottori University, Shimane Prefectural Central Hospital, Matsue Red Cross Hospital, Kurashiki Central Hospital, Tsuyama Central Hospital, Kawasaki Medical University, National Hospital Organization Okayama Medical Center, Okayama Red Cross Hospital, Hiroshima City Hiroshima Citizens Hospital, Hiroshima Prefectural Hospital, Hiroshima University, Tsuchiya General Hospital, National Hospital Organization Kure Medical Center, Yamaguchi University, Yamaguchi Prefectural Grand Medical Center, Tokushima University, Tokushima Municipal Hospital, Kagawa University, Shikoku Medical Center for Children and Adults, Matsuyama Red Cross Hospital, Ehime Prefectural Central Hospital, Kochi Health Sciences Center, Santamaria Hospital, National Hospital Organization Kyushu Medical Center, Kurume University, Kitakyushu City Hospital, University of Occupational and Environmental Health Japan, Fukuoka University, Kyushu University, Iizuka Hospital, National Hospital Organization Kokura Medical Center, Fukuoka Children's Hospital, National Hospital Organization Saga Hospital, Nagasaki University, National Hospital Organization Nagasaki Medical Center, Sasebo City General Hospital, Kumamoto City Hospital, Kumamoto University, Oita Prefectural Hospital, Almeida Memorial Hospital, Nakatsu Municipal Hospital, Miyazaki University, National Hospital Organization Miyakonojo Medical Center,

Kagoshima City Hospital, Imakiire General Hospital, Okinawa Prefectural Nanbu Medical Center & Children's Medical Center, Okinawa Prefectural Chubu Hospital, Naha City Hospital, and Okinawa Red Cross Hospital.
